# Burden, Outcome, and Comorbidities of Extrahepatic Manifestations in Hepatitis B Virus Infections

**DOI:** 10.3390/v16040618

**Published:** 2024-04-16

**Authors:** Busara Songtanin, Nattanicha Chaisrimaneepan, Roberto Mendóza, Kenneth Nugent

**Affiliations:** Department of Internal Medicine, Texas Tech University Health Sciences Center, Lubbock, TX 79430, USAkenneth.nugent@ttuhsc.edu (K.N.)

**Keywords:** extrahepatic manifestation, hepatitis B virus, hepatitis B infection, burden, morbidity, mortality

## Abstract

Hepatitis B virus (HBV) infections affect approximately 296 million people around the world, and the prevalence of any past or present HBV infection during the years 2015–2018 was as high as 4.3%. Acute HBV infection often presents with nonspecific symptoms and is usually self-limited, but 5% of patients can have persistent infections leading to chronic HBV infection and the risk of turning into chronic HBV infection is significantly higher in babies with vertical transmission (95%). Patients with chronic HBV infection are usually asymptomatic, but 15 to 40% of chronic HBV carriers develop cirrhosis and/or hepatocellular carcinoma. In addition to liver-related disorders, HBV is also associated with several extrahepatic complications, including glomerulonephritis, cryoglobulinemia, neurologic disorders, psychological manifestations, polyarthritis, and dermatologic disorders. Making the diagnosis of HBV can be challenging since patients with chronic infections can remain symptom-free for decades before developing cirrhosis or hepatocellular carcinoma, and patients with acute HBV infection may have only mild, nonspecific symptoms. Therefore, understanding how this virus causes extrahepatic complications can help clinicians consider this possibility in patients with diverse symptom presentations. The pathophysiology of these extrahepatic disorders likely involves immune-related tissue injury following immune complex formation and inflammatory cascades. In some cases, direct viral infection of extrahepatic tissue may cause a clinical syndrome. Currently, the American Association for the Study of Liver Diseases recommends treatment of chronic HBV infections with interferon therapy and/or nucleos(t)ide analogs, and this treatment has been reported to improve some extrahepatic disorders in some patients with chronic HBV infection. These extrahepatic complications have a significant role in disease outcomes and increase medical costs, morbidity, and mortality. Therefore, understanding the frequency and pathogenesis of these extrahepatic complications provides important information for both specialists and nonspecialists and may help clinicians identify patients at an earlier stage of their infection.

## 1. Introduction

Hepatitis B virus (HBV) infection is a significant global health problem, affecting approximately 296 million people worldwide. While the primary target of HBV is the liver, this viral infection can cause several extrahepatic disorders, affecting multiple organs and systems in the human body. These extrahepatic manifestations of HBV infection range from immune-mediated syndromes to direct viral effects, resulting in multiple clinical presentations, including renal disorders (glomerulonephritis, membranous nephropathy, membranoproliferative glomerulonephritis, IgA nephropathy), systemic manifestations (cryoglobulinemia, polyarteritis nodosa, serum sickness-like syndrome), and rheumatologic, hematologic, neurologic, ophthalmologic, and dermatologic disorders (Table 1) [1,2]. These extrahepatic complications can occur during both acute HBV infections and chronic HBV infections.

Even though the spontaneous rate of recovery in immunocompetent individuals infected with acute hepatitis B is as high as 95% [3], a small number of patients become chronically infected with HBV, defined as failing to clear HBsAg after 6 months, and can develop liver-related complications, including decompensated cirrhosis and hepatocellular carcinoma (HCC), which significantly increase mortality [3]. Understanding and recognizing these various presentations should improve diagnostic testing, management, and patient outcomes. This review article provides a comprehensive overview of the extrahepatic manifestations associated with HBV infection. By discussing the epidemiology, pathogenesis, clinical presentation, diagnostic approaches, and management strategies of these complications, this article should increase the knowledge and awareness of healthcare professionals of this important aspect of HBV infections. Overall, a better understanding of the extrahepatic manifestations of HBV infections can lead to more timely interventions and potentially improve outcomes for individuals with this chronic viral infection.

An overview of the pathogenesis of the extrahepatic disorders associated with HBV should include the following considerations. Hepatitis B virus is a hepatotropic virus which replicates in the liver. Viruses in the initial site of infection spread to adjacent hepatocytes resulting in an increased number of viruses in the liver and in the serum. This causes direct damage to hepatocytes and triggers complex immune responses. Extrahepatic involvement in this infection could reflect the consequences of immune-related injury or the consequences of extrahepatic tissue infection. Mason and coinvestigators have studied hepatitis B virus replication in diverse cell types during chronic infection [4]. In an early study in 1993, they analyzed HBV infection in four patients with chronic hepatitis who died [5]. Three of these patients had diffuse extrahepatic dissemination of virus based on the demonstration of nucleic acid sequences and proteins in the lymph nodes, spleen, bone marrow, kidney, skin, colon, stomach, and testes and neuroganglia. The involved cell types in these tissues included endothelial cells, macrophages, hematopoietic precursors, basal keratinocytes, mucosal epithelial cells, stromal fibroblasts, and neuronal cells. They subsequently studied two patients with extrahepatic involvement during HBV infection, one with polyarteritis nodosa and one with polymyositis [6]. Tissue samples from these two patients had detectable HBV RNA, replicative intermediates of HBV DNA, HBsAg, and HBcAg localized to the vascular endothelium. These findings would support the idea that some extrahepatic complications associated with HBV infection are related to virus infection in that particular tissue. Datta summarized relevant information in an editorial in 2015 [7]. This publication focused on peripheral blood mononuclear cells and noted that these cells support HBV infection, including replication, transcription, and production of infective virions. He also noted that HBV genome in peripheral blood mononuclear cells can undergo independent genetic evolution and develop drug resistance and that these infections have been transmitted through intrauterine routes during the pregnancy. Consequently, the pathogenesis of extrahepatic complications associated with HBV infections likely depends on the stage of infection, acute versus chronic, the degree or extent of liver injury, the immune responses associated with infection, including antibody formation, cellular immune responses, and immune complex formation, and direct tissue infection by the virus. Infections in extrahepatic tissue have potential consequences which could include viral genetic evolution, the development of drug resistance, and the provision of a low-level viral reservoir which has the potential to reinfect the liver. These possibilities will be discussed in subsequent sections when information is available pertinent to the particular organ system.

## 2. Systemic Diseases

### 2.1. Cryoglobulinemia

Immunoglobulins in the serum that precipitate at temperatures below 37 °C are called cryoglobulins. The classification of cryoglobulinemia includes three types; type II and type III are also referred to as mixed cryoglobulinemia. Mixed cryoglobulinemia has a strong association with hepatitis C virus (HCV) infection in nearly 90% of cases; however, HBV infection has also been associated with cryoglobulinemia. The prevalence of HBsAg in cryoglobulinemia varies between 1.8% to 5.5% [8,9]. A retrospective study by Han included 23 patients with HBV-related cryoglobulinemia showed that clinical manifestations vary widely in patients and include purpura (65.2%), renal function impairment (43.5%), peripheral neuropathy (21.7%), livedo reticularis (8.7%), and several renal disorders, including proteinuria (69.6%), hematuria (39.1%), and glomerulonephritis, specifically, membranoproliferative glomerulonephritis (MPGN) similar to HCV [10]. Similar to HCV, type I MPGN is the most common renal manifestation in HBV-related cryoglobulinemia [11]. Other system involvement includes joints (8.7%), gastrointestinal tract (4.3%), and cardiovascular system (4.3%). Patients can also develop a severe form of cryoglobulinemia with end-organ damage, including vasculitis in the heart, gastrointestinal tract, and central nervous systems [2]. Mixed cryoglobulinemia can sometimes evolve into low-grade non-Hodgkin lymphoma (discussed later in the hematologic section).

The underlying mechanism includes (1) circulating immune complexes formed by HBV, polyclonal IgG, and monoclonal IgM that are deposited on the endothelial surfaces causing vascular inflammation, systemic inflammation, tissue damage, and organ dysfunction; (2) chronic antigenic stimulation by HBV leads to the expansion of B-cells from polyclonal B-cell to oligoclonal B-cell expansion and is a transitional phase between autoimmunity and neoplasm [12,13].

The treatment of HBV-related cryoglobulinemia is based on the severity of cryoglobulinemia (mild, moderate to severe, and life-threatening). The medications and management used in the treatment include: (1) antiviral therapy with nucleoside analogs (Entecavir and Tenofovir) that remain the first-line treatment for HBV-associated cryoglobulinemia, with the goal of suppressing HBV replication and are commonly used in mild to moderate disease. A study showed that the decrease in viral replication was associated with the serum clearance of cryoglobulin and normalization of rheumatoid factor; (2) immunosuppressive therapy, e.g., glucocorticoid to control the recurrence of vasculitis; (3) removal of circulating cryoglobulins with plasma exchange is considered the most efficient way to improve acute conditions in patients with severe cryoglobulinemic vasculitis. Corticosteroids and plasma exchange can be co-administered to control severe cryoglobulinemic vasculitis flares; (4) B-cell depleting therapy with Rituximab (a chimeric monoclonal antibody to anti-CD20 antigen) to eliminate B lymphocytes that produce cryoglobulins. Several studies have demonstrated the effectiveness of Rituximab in treating cryoglobulinemia in patients who do not respond to the antiviral therapy alone; (5) anti-inflammatory drugs (NSAIDs) [10,13,14,15].

In summary, in patients with a mild form of cryoglobulinemia (purpura, arthralgia), antiviral monotherapy can be useful [2]. In cases of severe cryoglobulinemia (glomerulonephritis, neuritis, necrotizing skin ulcers), Rituximab alone or with glucocorticoids is recommended. The combination of plasma exchange and glucocorticoids should be considered in life-threatening cases. The management of patients with cryoglobulinemia suggests that the pathogenesis involves aberrant immune responses. The treatment of these deleterious immune responses has the potential to change the natural history of this viral infection. The risk of reactivation of hepatitis B is as high as 25% in patients with past HBV infection (anti-HBc Ab positive, HBsAg negative); in HBsAg-positive patients who are not receiving antiviral therapy, up to 85% can have a hepatitis flare [16].

### 2.2. Polyarteritis Nodosa

Polyarteritis nodosa (PAN) is a medium-sized arterial vessel disease that causes systemic necrotizing vasculitis; it can also affect small-sized arterial vessels. Polyarteritis nodosa can occur in both acute and chronic HBV infections. It presents in 1 to 5% of HBV infections and usually occurs in the first six months of infection [2,17]. Thirty-five to fifty percent of the patients with PAN are positive for HBsAg. Clinical manifestations include polyarthritis, arthralgia, rash, fever, abdominal pain, diarrhea, weight loss, peripheral neuropathy, hypertension, and renal failure; these complications are similar to those seen with idiopathic PAN. However, patients with HBV-associated PAN can have a more severe involvement of the gastrointestinal tract with gastrointestinal bleeding and perforation. The pathophysiology is not well understood but may be caused by virus-induced immune dysregulation which leads to endothelial damage and subsequent formation of immune complexes that deposit in the vessels, ultimately resulting in vasculitis [18]. The affected vessels become thickened secondary to intimal proliferation which leads to decreased blood flow and organ perfusion. One case report has documented the presence of virus in the endothelial cells in these patients [6]. 

Treatment includes antiviral therapy before immunosuppressive medications. Patients with severe hepatitis B-associated PAN may require glucocorticoids and plasma exchange in addition to antiviral drugs [19].

### 2.3. Serum Sickness-like Syndrome

Serum sickness is an immune complex-mediated hypersensitivity reaction that typically occurs following exposure to foreign proteins, such as drugs or biological agents. A transient serum sickness-like syndrome develops in about 10 to 20% of patients with acute HBV infection [1]. Hepatitis B vaccine, which contains viral proteins, has also been associated with the development of serum sickness in rare cases [20]. The clinical presentation varies widely and includes fever, rashes (erythematous maculopapular or macular rash, urticarial, nodular, petechial), and polyarthritis with laboratory findings of low levels of complement [1,21]. The symptoms can occur before the onset of viral symptoms of acute HBV infection [2]. Pathophysiology includes circulating immune complexes which are composed of HBsAg which leads to the consumption of complement. In cases of HBV vaccine-related serum sickness, the pathophysiology probably involves the formation of immune complexes following the interaction between the vaccine antigens and preexisting antibodies in susceptible individuals. Currently, there is no established treatment, and symptoms tend to regress with the onset of the symptoms of hepatitis. Supportive treatment usually leads to the resolution of symptoms.

## 3. Hematologic Complications

Lymphoproliferative diseases are disorders characterized by abnormal growth and accumulation of lymphocytes, leading to impaired immune responses. Chronic HBV infection can dysregulate host immune responses and increase the risk of developing non-Hodgkin lymphoma (NHL) and Hodgkin lymphoma. Several mechanisms have been proposed to explain this association, including chronic antigen stimulation, direct viral oncogenic effects, impaired immune surveillance, and viral integration into the host genome. Hepatitis B virus can infect peripheral blood mononuclear cells, and this possibly triggers lymphoproliferation and malignant transformation.

### 3.1. Lymphoma

The prevalence of HBV in non-Hodgkin lymphoma (NHL) ranges from 6.9 to 30.8% [22,23]. The B-cell subtype of NHL has a prevalence of up to 30.2% [24]. Several studies have reported that patients with HBV-associated NHL tend to be younger (46 years vs. 51) and have more advanced disease compared to those with non-HBV-associated NHL. Ulcickas et al. reported that patients with HBV infection were 2.8 times more likely to develop NHL than patients without HBV infection [25]. Diffuse large B-cell lymphoma (DLBCL) is the predominant type in HBV-associated NHL [26,27,28]. Other hematologic diseases, including B-cell NHL, multiple myeloma, and acute lymphoblastic leukemia, also have a higher frequency in patients with HBV infections compared to patients without HBV infection. The risk of aggressive B-cell NHL, including DLBCL, mantle cell lymphoma, primary mediastinal large B-cell lymphoma, and Burkitt lymphoma, is also significantly higher [29]. A meta-analysis by Li et al. showed that within the B-cell NHL subtypes, HBV infection was significantly associated with DLBCL and follicular lymphoma but not with chronic lymphocytic leukemia, small lymphocytic lymphoma and Burkitt lymphoma [30]. Treatment with chemotherapy or immunotherapy, especially with anti-CD20 monoclonal antibody, in NHL can cause HBV reactivation in previously infected patients and will not be discussed in this review [31].

The underlying pathophysiology of HBV-associated NHL is not well established, unlike HCV-associated NHL. The proposed hypotheses include both direct and indirect mechanisms [32]. The direct mechanism is primarily driven by the oncogenic effect mediated by intracellular HBV DNA; the indirect mechanism occurs from chronic stimulation of B-cell lymphocytes by extracellular HBV. The hypothesis that HBV infection may cause transformation of B cells is supported by two studies. Wang et al. detected HBV antigen and DNA in diffuse large B-cell lymphoma (DLBCL) tissue, providing direct evidence for this hypothesis [33]. Ren et al. found that HBsAg-positive DLBCL is an antigen-independent mechanism rather than a chronic antigenic stimulation [28]. The study by Wang et al. also showed lower numbers of anti-HBs-positive and anti-HBc-negative patients in NHL cases, suggesting that anti-HBs without anti-HBc response may effectively control HBV replication in lymphoid cells and prevent neoplastic transformation and the development of NHL [24]. 

The main therapy for HBV-associated NHL involves management with immuno-chemotherapy (Rituximab-CHOP or R-CHOP-like regimen for DLBCL) and antiviral treatment. One study reported a complete response in 67% of patients following antiviral and immuno-chemotherapy [34].

### 3.2. Aplastic Anemia 

Aplastic anemia is a disease caused by immune-mediated destruction of hematopoietic stem cells, resulting in pancytopenia and a paucicellular or fatty bone marrow. Hepatitis-associated aplastic anemia (HAAA) is a variant of aplastic anemia in which bone marrow fails to function following hepatitis [35]. Several hepatitis viruses, including hepatitis A virus, HBV, HCV, hepatitis E, and hepatitis G, have been reported to cause HAAA [36]. Hepatitis-associated aplastic anemia occurs predominantly in children, adolescent boys, and young men. Clinical manifestations vary widely and include spontaneous mucosal or cutaneous bleeding due to thrombocytopenia, an increased risk of bacterial or fungal infection due to neutropenia, fatigue, and pallor due to anemia with a low reticulocyte count. Intracranial hemorrhage, although a very rare complication, is fatal [36]. Symptoms of HAAA usually begin during the recovery phase of acute hepatitis [36,37]. Pancytopenia can occur concomitantly or 2–3 months and up to 12 months after acute hepatitis [35]. Liver dysfunction tends to improve when the pancytopenia develops [38]. Other laboratory findings that may help establish the diagnosis of HAAA include hypogammaglobulinemia, a low CD4/CD8 T cell ratio, an increased number of cytotoxic cells, and a bone marrow biopsy that generally demonstrates hypocellularity affecting all cell lines and is replaced by fat cells with no infiltration or fibrosis [35,36,39].

Cytotoxic T lymphocytes (CTLs, CD 8) possibly contribute to the pathogenesis of aplastic anemia and bone marrow destruction. During the early phase of hepatitis, cytotoxic T lymphocytes recognize similar antigens of the liver and bone marrow cells [40,41]. Afterward, the CTL clonal expansion leads to bone marrow hematopoietic stem cell destruction, resulting in aplastic anemia. A study by Kagan et al. and another study by Cengiz et al. reported that patients with aplastic anemia had a decreased ratio of CD4/CD8 cells and a high percentage of CD8 cells which appeared to be cytotoxic and myelopoietic in vitro [42,43]. The finding of low CD4/CD8 was also found in HAAA compared to non-hepatitis-associated aplastic anemia, which could help predict the development of HAAA [44,45]. The CD8 Kupffer cells detected in liver biopsies in HAAA patients may be the mediator of this condition [38].

Treatment of this disorder includes both supportive treatment and definitive treatment. Supportive treatment comprises specific blood product transfusion. Red blood cells and platelet units should be leukocyte-reduced to minimize the risk of cytomegalovirus transmission [36]. Patients who are candidates for hematopoietic stem cell transplantation should receive irradiated and leukocyte-depleted units to prevent the risk of future transfusion-associated graft-versus-host disease. The definitive treatment of HAAA includes immunosuppressive therapy and allogeneic bone marrow transplant. Anti-thymocyte globulin and cyclosporine are associated with a low risk for hepatitis B reactivation in patients with HAAA. Corticosteroids have also been used in combination with immunosuppressive agents for the treatment of the HAAA patients [46]. HBV DNA levels and liver enzymes should be closely monitored while receiving immunosuppressive therapy, and concomitant therapy for HBV infection is recommended. The 5-year overall and failure-free survival in patients following allogeneic hematopoietic cell transplantation is as high as 86% [47]. Delays in the treatment and age of onset are the factors that affect outcomes. However, most of the patients with aplastic anemia who survive have complete recovery [48].

## 4. Renal Disease

Glomerular disease in association with HBV was first described by Combes et al. in 1971 when a patient with post-transfusion antigenemia developed membranous glomerulonephritis, with granular staining of IgG and C3 using immunofluorescence [49].

### 4.1. Pathogenesis

Renal disease associated with HBV infection occurs mostly in endemic regions and often develops when transmission occurs during infancy or early adolescence, which increases the probability the individual will become a chronic carrier [50]. The pathogenesis primarily consists of hepatitis B antigen–antibody complex accumulation in the renal lesions identified by immunofluorescence microscopy [51]. The current hypothesis is that the low molecular weight of HBeAg at 300 kDa allows the subunit to pass through the glomerular basement membrane, resulting in the formation of immune deposits; however, whether a specific genotype contributes to a different extent than others remains unclear [52]. Recent studies demonstrate that the pathogenesis of HBV glomerular disease is directly related to the virus and purified HBV can directly stimulate the proliferation of human mesangial cells and the expression of type IV collagen and fibronectin [53]. Moreover, elevated viral DNA concentrations increase the possibility of antigen deposition which can cascade to activation of various inflammatory cells and cytokines following immune complex formation [54]. 

The possible pathophysiology could involve (1) a humoral response triggering the HBAg-HBAb immune complexes from a potential immune cellular defect, or (2) HBV-infected renal cells resulting in HBcAg expression that could activate T-lymphocytes to release lymphokines that could increase glomerular permeability, or (3) a combination of these processes. Following a single biopsy, patients usually have one type of histologic lesion or have concomitant features of several types of glomerulonephritis; however, the dominant histology likely depends upon the qualitative and quantitative differences in the immune complexes present in the kidney [55]. 

### 4.2. Treatment

HBV-associated glomerulonephritis (HBV-GN) treatment principally focuses on (1) reducing proteinuria, (2) treating and preventing recurrence, and (3) protecting renal function and delaying the progression of renal disease. Antiviral treatment is the main approach for treating these renal manifestations with entecavir being the first-line drug due to its high efficacy and the infrequent development of drug resistance [56]. Even so, antiviral monotherapy has its limitations with HBV mutations, low remission rates, and long time to remission. Wang et al. have previously shown in a cohort of 42 hospitalized patients with HBV-GN between the ages of 18 to 70 years that tacrolimus-entecavir combination therapy significantly reduced proteinuria without increasing the viral DNA load or causing acute renal function deterioration during combination therapy or later with entecavir monotherapy [57,58]. Although promising, larger studies are needed to establish this result since Wang et al. used a similar regimen in two small groups with a one-year follow-up and did not find a significant difference in the proteinuria remission rates [59].

### 4.3. Membranous Nephropathy

Membranous nephropathy (MN), also called membranous glomerulopathy, is the second most common nephropathy in adults, is the most common extrahepatic renal manifestation of HBV infection, and is the most common pathological category of HBV-glomerulonephritis [60]. It is characterized by diffuse thickening of arterial segments and glomerular basement membrane on light microscopy [61]. Both primary and HBV-associated MN (HBV-MN) can further be identified with its pathognomonic “spike and dome” appearance of subepithelial deposits on electron microscopy; however, mild mesangial proliferation may also occur in HBV-MN [61]. In a study analyzing the pathologic features in HBV-MN and idiopathic MN, glomerular disease secondary to HBV infection had more deposition of IgG1 and C1q and less IgG4 in the HBV-MN group than in the idiopathic MN [59].

Patients with HBV-MN present with massive proteinuria, often exceeding 3.5 g/day, due to podocyte injury and loss of membrane anionic charge, resulting in albuminuria. The clinical manifestations resemble idiopathic MN and include peripheral edema, hypertension, frothy urine, and thromboembolic events. Laboratory analysis reveals hypoalbuminemia, dyslipidemia, and acute kidney injury with elevated creatinine levels. The disease usually has a benign course in children and has a male predominance in up to 80–100% in HBV-infected children. Younger patients and those with minimal subepithelial immune deposits have a greater likelihood of remission of MN within four years [62,63]. Pediatric patients often experience spontaneous remission of proteinuria within one year of diagnosis, accompanied by preserved renal function and seroconversion from HBeAg to anti-HBe. Spontaneous recovery to baseline is relatively uncommon in adults, and some patients may have renal deterioration over time [64]. Lai et al. studied 21 patients with adult-onset HBV-MN and found that patients infected in endemic areas had infrequent spontaneous remission. Twenty-nine percent had a slow decline in renal function and 10% developed end-stage renal disease during a 5-year follow-up period [51].

### 4.4. Treatment

Several studies have recommended antiviral monotherapy for HBV-related glomerulonephritis to decrease urine protein excretion. A meta-analysis conducted by Yang reported that antiviral therapy effectively induces proteinuria remission and HBeAg clearance in HBV-MN patients, with no significant difference found between interferon and nucleoside analogs [60]. Immunosuppressive drugs and corticosteroids have been used in some case reports; however, these drugs can potentially trigger transient viral replication, leading to increased HBeAb and HBV-DNA levels. Moreover, these agents do not reduce proteinuria, promote HBeAg clearance, or improve kidney lesions.

### 4.5. Membranoproliferative Glomerulonephritis

Membranoproliferative glomerulonephritis (MPGN) is a nephritic syndrome (defined as the presence of hematuria, proteinuria, hypertension, decrease urine output, and edema) and thus involves an inflammatory injury of glomerular basement membranes, that often co-presents with nephrotic syndrome (defined as the presence of heavy proteinuria with the protein excretion greater than 3.0 g/24 h, hypoalbuminemia, and peripheral edema) and has mesangial expansion with capillary wall thickening that results in lobuloid glomerular tufts on light microscopy [65,66]. Furthermore, the capillary wall double-contours into a “tram-track” appearance with hypercellularity and interpositioning of neutrophils and monocytes. Deposits of immune complexes containing IgG, complement components, and IgM in the subendothelial, mesangial, and paramesangial areas can also appear granular in the pattern under immunofluorescence. Hepatitis B virus infection has an association with type I and type III MPGN. 

The clinical presentation of MPGN varies from asymptomatic hematuria and proteinuria to nephrotic and nephritic syndrome and can even progress to rapidly progressive crescentic glomerulonephritis. Renal function may reveal a normal or elevated creatinine, and hypocomplementemia is likely present in all types of MPGN. The histopathology of HBV MPGN demonstrates the deposition of antigen–antibody complexes which mainly consist of IgG and C3 in the mesangium and subendothelial space [67].

### 4.6. Treatment

Unlike HBV-membranous nephropathy, the treatment of HBV-associated membranoproliferative glomerulonephritis has not been well established; therefore, the efficacy of antiviral monotherapy for HBV-MPGN remains unknown [68]. One case report involved a 28-year-old postpartum woman who had renal biopsy confirmed MPGN type III and was initially started on entecavir at a dose of 0.5 mg once daily [68]. Even though the HBV DNA became undetectable after 1 month of treatment, her kidney function did not improve as demonstrated by persistent proteinuria after 7 months of entecavir with a repeat biopsy showing evidence of active MPGN. Corticosteroid treatment was started with an initial dose of prednisolone 30 mg/day with a schedule for tapering; 7 months after prednisolone was started and tapered to 5 mg/day, the third renal biopsy demonstrated decreased activity of HBV-MPGN with reduced mesangial proliferation and immune complex deposition.

### 4.7. IgA Nephropathy

IgA nephropathy is the most common glomerular disease worldwide. It is an immune complex-mediated glomerulonephritis with significant IgA deposition in the mesangium of the glomerulus [69]. The pathogenesis of IgA nephropathy remains unclear. One systematic review of biopsy-based studies from several countries reported a general population incidence of at least 2.5 per 100,000 [70]. IgA nephropathy can be histologically identified by mesangial proliferation on light microscopy and IgA-based immune complex deposition in the mesangium with both immunofluorescence and electron microscopy.

Hepatitis B virus infection is one of several possible causes of IgA nephropathy. There have been conflicting reports as to whether HBsAg is directly responsible for the pathogenesis of IgA nephropathy or indirectly responsible for initiating an underlying cascade [67,71,72]. Wang et al. studied eight patients with IgA nephropathy and reported that HBV DNA was detectable by in situ hybridization with a rate of 100%; five out of the patients were also HBV DNA positive by Southern blot analysis and all had integrated forms which reflects viral transcription in glomerular cells and renal tubular epithelia [73]. This is consistent with transgenic mice studies that revealed the expression of the viral genome of HBcAg or HBeAg only in epithelial cells [74,75]. Therefore, similar to the liver, the kidneys may serve as a reservoir for HBV DNA after viral infection. Wang et al. recently found in a single-cohort retrospective study with 838 patients with biopsy-confirmed IgA nephropathy that HBV served as an independent risk factor for IgA nephropathy with a significant difference in renal survival between primary and HBsAg IgA nephropathy after a 2.4-year follow-up. Patients with primary IgA nephropathy had a better prognosis than those with HBsAg IgA nephropathy [59]. 

Since IgA nephropathy is a nephritic syndrome, patients often present with asymptomatic hematuria that usually follows a recent respiratory or gastrointestinal infection with IgA secretion by mucosal linings. Progressive chronic kidney disease also frequently accompanies this condition, but renal survival can vary depending on biopsy timing and introduction of lead-time bias. Clinical findings associated with a poor prognosis include hypertension, proteinuria, and decreased eGFR at diagnosis [76].

Renin-angiotensin-aldosterone system inhibitors have been the standard therapy when treating IgA nephropathy patients and can be combined with corticosteroids and other immunosuppressive drugs for patients at high risk of disease progression [77,78]. However, because of the etiologic agent in HBsAg IgA nephropathy patients, antiviral drugs should be included in the immunosuppressive regimen. A prospective study in China reported that the use of lamivudine in combination with prednisolone (19/29 = 65.5%) resulted in a higher rate of proteinuria remission compared with prednisolone only therapy (9/17 = 52.9%) in adult patients with inactive HBV carriers (normal liver enzymes, HBsAg positive, HBeAg negative, and HBV-DNA negative) with biopsy-proven IgA nephropathy with a follow-up period of 18 months with a *p*-value of 0.399 [79]. This study defined complete remission of proteinuria in that protein < 0.5 g/day. In addition, none of the patients in the lamivudine and prednisolone had evidence of HBV reactivation defined as detectable serum HBV-DNA or HBeAg and increased alanine aminotransferase (ALT) levels (>120 U/L). HBV reactivation and significant ALT elevation occurred in 3/17 (17.65%) of patients in the monotherapy group (*p* = 0.019).

## 5. Neurologic Disease

### 5.1. Guillain-Barré Syndrome

One of the most common immune-mediated demyelinating polyradiculoneuropathies, Guillain–Barré syndrome (GBS) is an eponym that includes various clinical presentations of this disorder. While *Campylobacter jejuni*-related GBS is a relatively straightforward diagnosis, with one meta-analysis attributing up to 30% of GBS cases to this Gram-negative bacterium, and good evidence supporting its molecular mimicry pathophysiology, several microorganisms can initiate this disease process due to similar cross-reactivity of shared epitopes that could have an atypical, and often missed, clinical presentation [80,81]. About 1% of GBS cases have been found to be associated with acute hepatitis (A, B, C, D, and E) [82]. The association between HBV infection and GBS has been reported in case studies, but the underlying pathophysiology is not well described. For example, a 42-year-old woman presented to the hospital with 2 weeks of a viral prodrome and was found to have acute HBV infection. She developed rapid weakness and paralysis in all extremities 4 days after hospitalization and was diagnosed with GBS confirmed with lumbar puncture, electromyography, and nerve conduction studies. The patient was treated with intravenous immune globulin (IVIG) without antiviral therapy and recovered completely 6 weeks after the onset of neurologic symptom [83]. 

Some proposed effectors in its mechanism include molecular mimicry between HBV DNA and myelin basic protein with host immunity attacking both, direct damage by HBV, or HBsAg-mediated immune complex vasculitis [83]. Not only acute HBV infection is associated with GBS, but also cases of chronic HBV infection have been reported [82]. Wei reported a 34-year-old man who had a history of chronic HBV infection diagnosed 2 years prior to admission who developed GBS confirmed with nerve conduction study and lumbar puncture. This complication developed during an acute exacerbation of chronic HBV infection with elevation in liver enzymes from baseline (AST from 231 to 723 IU/L, ALT from 204 to 533 IU/L). The patient received IVIG along with tenofovir disoproxil fumarate 300 mg/day orally for 5 days but deteriorated with worsening respiratory symptoms requiring tracheostomy associated with persistent elevation of liver enzymes. The decision was made to administer a second dose of IVIG; the patient’s condition improved, and he was discharged home on Day 29. Tsukada et al. published a case series that included four patients with GBS-associated with HBV infection and reported a significant increase in HBsAg-immune complexes (IgG and C3 complement) in the serum and cerebrospinal fluid. The level of immune complex was closely related to the clinical status of the patients [84]. The study also found a positive correlation of the HbsAg immune complex as demonstrated by electron-dense deposits around the endo-neural small blood vessels and in the endoneurium. The study could not conclude whether the immune complexes were formed in situ or that circulating immune complexes were deposited in the endoneurium. In addition to an association between acute and chronic HBV infection and GBS, a case of GBS following HBV vaccination has been reported in a patient who developed GBS 10 weeks after receiving recombinant HBV vaccine [85]. 

The main therapy for HBV-associated GBS includes IVIG with antiviral drugs and has been demonstrated to improve the outcome with complete recovery [82]. The antiviral therapy discussed in the literature includes nucleoside analogs (lamivudine, entecavir, and tenofovir disoproxil fumarate) and should be started early in the disease course as the medication may inhibit HBV replication and reduce the formation of HBsAg immune complexes resulting in damage to nerves [82]. 

### 5.2. Vasculitic Neuropathy and Non-Vasculitic Neuropathy

Neuropathies caused by vasculitis are a heterogeneous group of disorders that may be associated with systemic vasculitis or localized to peripheral nerves, though the latter occurs less frequently. These disorders have an acute to subacute onset of sensory dysfunction (pain) and motor deficits that result from inflammatory destruction of the vessels and subsequent ischemia of the nerve tissue [86]. Several classifications, including the Peripheral Nerve Society Task Force and the International Chapel Hill Consensus Conference, describe the various neuropathies associated with vasculitis; also, a distinction based on the size of the involved vessel is useful [87,88]. Vasculitis is the major pathology in HBV-related neuropathy characterized by the deposition of immune complexes containing viral antigens into the endothelium of vasa nervorum in the epineurium along with perivascular lymphocytic infiltrates [89]. In most cases, this vasculitis develops in the acute stage of HBV infection [90]. However, patients who remain HBsAg-positive for ≥6 months are considered hepatitis B carriers, and this may also result in neuropathy with relapses during a chronic infection [91]. Patients may clinically present with the sudden onset of proximal deep aching pain in the limb, followed by burning cutaneous pain and focal weakness in the territory of a single nerve [92]. Within a month, this presentation can progress to a symmetrical or asymmetrical distal localized polyneuropathy [93]. Approximately 30% patients with vasculitic neuropathy present with a subtle but progressive disease course. 

Non-vasculitic neuropathy associated with HBV has been rarely reported [94,95]. A case report described a patient with chronic HBV infection who presented with sensorimotor mononeuropathy multiplex; these symptoms improved with oral prednisone and the antiviral drug lamivudine for 2 months, and HBV DNA levels decreased which suggests chronic immune-mediated, non-vasculitic mononeuropathy multiplex and viral replication caused disease activity [94]. The underlying pathophysiology in neuronal damage in both acute and chronic HBV infection remains uncertain; hypotheses include (1) immune-mediated neuronal damage secondary to the direct action of the virus on the nerve fibers, or (2) deposition of immune complexes of HBsAg and HBeAg in the vasa nervorum, and (3) viral replication in association with disease activity, as suggested by the high titers of HBV DNA and the improvement in clinical symptoms after the administration of antiviral drugs. Because HBV-associated vasculitic neuropathy occurs commonly with a mixed cryoglobulinemia presentation, the preferred treatment includes entecavir for antiviral therapy, started concomitantly with glucocorticoids and rituximab, regardless of HBe and HBV DNA status [96,97]. An exception to rituximab use is the patient in an active hepatitis flare since this situation can have fatal consequences [98].

## 6. Ophthalmologic Disease 

### 6.1. Uveitis

Uveitis is inflammation of the uvea, a vessel-rich layer of the eye positioned between the sclera and retinal pigmented epithelium, which consists of the choroid, ciliary muscle, and iris. Because of this vascularity, distant activation of the immune system can cause hematogenous spread of cytokines and inflammatory cells that result in uveal inflammation. In Western countries, uveitis causes up to 20% of legal blindness, and uveitis causes approximately 25% of blindness in developing nations [99] The study of uveitis in association with hepatitis demonstrates a significant higher risk of uveitis in patients with HBV and HCV coinfection (hazard ratio [HR] = 2.88, 95% CI = 1.07–7.78) [100]. A study in Switzerland reported that 13% of patients with uveitis were sera-positive for HBsAg [101]. Fraunfelder et al. has collected 32 reports of uveitis that occurred after HBV vaccination [102]. 

The mechanism of HBV-associated uveitis remains largely unknown; several studies have reported the presence of HBsAg in tears and aqueous humor in seropositive HBsAg patients [103]. The possible pathophysiology of HBV-associated uveitis includes the complement-mediated immune pathway in the retinal vasculature leading to the release of inflammatory mediators causing uveoretinal inflammation indicating that autoimmunity contributes to the development of these diseases. Some animal studies have found that HBV DNA polymerase has similar amino acid sequences with uveitopathogenic epitopes [104]. Others suggest that HBV can serve as an environmental trigger for the development of uveitis, but the information supporting this hypothesis remains insufficient at this time [105].

The symptoms of uveitis depend on its specific location. Anterior uveitis, or iritis, can present with erythema at the limbus, the junction between the cornea and the sclera, and these patients often have miotic pupils and pain. Upon slit lamp examination, detection of leukocytes in the anterior chamber is sensitive for anterior uveitis but is non-specific. Posterior or intermediate uveitis, however, is confirmed by visualization of active chorioretinal inflammation with possible leukocytes in the vitreous humor. In more developed cases, panuveitis develops, further increasing the risk for complications that include band keratopathy, posterior synechiae, cataract formation, intraocular hypertension, and cystoid macular edema. Treatment efficacy has not been firmly established but should include antiviral therapy and supportive care based on symptoms [106]. 

### 6.2. Optic Neuritis

HBV-associated optic neuritis is the main component of neuromyelitis optica spectrum disorder (NMOSD) post infection; other criteria include a T2 MRI lesion extending over three vertebral segments, in association with brain MRI findings inconsistent with multiple sclerosis [107]. Furthermore, optic neuritis associated with HBV infection has unique resistance to conservative doses of oral corticosteroids after vision loss, and higher doses are needed to restore vision in patients [108]. This is a stark contrast to the typical optic neuritis case which presents with mild to moderate vision impairment, usually uniocular. Moreover, whereas typical optic neuritis has maximum loss of vision within 2 weeks after onset, vision loss from autoimmune optic neuritis after HBV infection can extend beyond this time period. Heekin et al. also reported a case after immunization against HBV [109]. This HBV complication has remained mostly unexplored with an unclear pathophysiology. The simultaneous development of elevated levels of HBV antigens and AQP4-IgG are possibly involved in the pathogenesis.

## 7. Rheumatologic Diseases

Extrahepatic rheumatological complications occur as a result of autoreactive lymphocytes and circulating autoantibodies, or immunoglobulins reacting against self-antigens, leading to autoimmunity [105,110,111]. The combination of genetic and environmental factors contributes to the development of autoimmunity. Some viral infections, including human herpesvirus, HIV, parvovirus B19, and HBV, can activate autoreactive T cells and cause virus-induced tissue injury [112]. 

Rheumatic complaints may be the first symptoms in patients with clinically silent HBV infection. Due to the wide clinical spectrum of rheumatic complaints in hepatitis infection and the high frequency of rheumatic complaints in the general population, this presentation can lead to a delayed diagnosis of HBV infection and increase the risk of complications. A cross-sectional study by Oliveira et al. found that 19 patients out of 38 patients with HBV infection had nonspecific rheumatic complaints, which were not consistent with any specific rheumatic disease, probably as a consequence of viral infection [113].

### 7.1. Rheumatoid Arthritis

Rheumatoid arthritis is an autoimmune inflammatory disease characterized by symmetrical inflammation and destruction of joints [114]. The contribution of genetic, infectious, environmental, and hormonal factors has been postulated, but the true etiology of RA remains unclear [105,115]. Viruses have been reported as a cause of arthritis, and there is an association between HBV and RA. One possible explanation is that viral infection leads to interleukin-6 and tumor necrosis factor-α production, which contribute to the induction and maintenance of RA [105]. Another proposed mechanism is molecular mimicry which leads to the production of autoantibodies [116]. Whether viruses are actual causative agents of RA has not been fully determined.

Patients with chronic HBV can have arthralgia or arthritis similar to RA, and the diagnosis of RA, regardless of any association with HBV infection, requires patients to test positive for both RF and antibodies against cyclic citrullated peptide (anti-CCP) [117]. Lim et al. recruited 240 patients and found that the prevalence of RA in patients with chronic hepatitis B was approximately 4% [118]. Rheumatoid factor was detected more frequently in asymptomatic carriers of HBV surface antigen (HBsAg) than in healthy individuals (11.8% vs. 3.4%). Rheumatoid factor positive rates were not significantly associated with the level of alanine aminotransferase or C-reactive protein in individuals with HbsAg, but HBV DNA levels did correlate with rheumatoid factor (RF) titers. Antibodies against cyclic citrullated peptide (anti-CCP) were detected in individuals with HBsAg less often than was RF (4.6% vs. 11.8%). In patients with HBsAg, RF was present in 46% of patients with arthritis, 5% of patients with arthralgia, and in 8% of patients without rheumatologic complaints. Anti-CCP was present in 36%, 0%, and less than 1% of these patients’ categories, respectively. Joint disorders and biochemical changes classified as rheumatoid arthritis were found in 4.1% of patients with HBsAg. These patients accounted for 32.1% of HBV infected individuals with RF, 81.8% of those with anti-CCP, and 90% with both RF and anti-CCP. Senna et al. recruited 219 patients and found true rheumatoid arthritis related to HBV infection in 4% of the cases [119]. However, Feuchtenberger et al. studied 79 patients and detected HBV infection in only 0.2% of patients who presented with RA; in a nationwide database study by Hsu et al. with 3320 patients, HBV infection was detected in 9.3% of Chinese RA patients [120,121]. A study by Zou et al. suggested that the presence of HBV in RA synovium might be involved in the pathogenesis of synovitis and exacerbate disease progression in RA [122]. All these studies support the possibility that HBV can have a pathogenic role in RA.

### 7.2. Non-Rheumatoid Arthritis

Musculoskeletal symptoms are among the most common extrahepatic symptoms in patients with HBV. In HBV carriers, the frequency of arthralgia ranges from 3% to 53%, myalgia occurs in 3% to 58%, and arthritis occurs in fewer than 2% [123,124]. The underlying mechanism of non-rheumatoid arthritis is the intra-synovial deposits of circulating immune complexes in the small joints activated by the complement cascades containing HBsAg-anti-HBs antibodies, immunoglobulins, and complement components [1,123]. 

Arthritis can occur during the pre-icteric phase of acute hepatitis B infection, when a patient has flu-like symptoms, or during the chronic phase of the infection. The arthritis can be migratory or additive or can involve all affected joints simultaneously [125]. The most affected joints are metacarpophalangeal and proximal interphalangeal joints [126]. Monoarthritis of a large joint was found in about 40% of cases, polyarthritis of the small joints of the hands in 10%, and a combination of large and small joint involvement in 50% [127]. The knees are the most commonly affected large joints, followed by wrist, ankle, elbow, shoulder, and hip joints. In a review by Maslennikov et al., cervical and lumbar intervertebral joints were involved in about 10% of cases and usually occurred together with other joints [127]. The pattern can be symmetrical or asymmetrical, but more commonly symmetrical. Morning stiffness with muscle aches may mimic an acute attack of rheumatoid arthritis [126].

During the acute infection, arthritis is abrupt in onset and presents primarily or following a prodrome of malaise and anorexia [125]. Synovitis develops abruptly and is severe. Arthritis develops within 12 week after the onset of the disease, but this interval ranges from 6–20 week [127]. In approximately 20% of acute HBV infections, arthritis occurs 1–6 weeks before hepatitis [128]. The duration of arthritis before jaundice has a mean duration of two and a half weeks. However, arthritis following the acute prodromal infection may recur intermittently or persist indefinitely [129]. 

The arthralgias associated with chronic hepatitis characteristically involve multiple joints with an asymmetrical pattern and are associated with asymmetrical polyarthritis with erythematous skin lesions [105]. A study by Inman concluded that in rheumatic complications associated with HBV infection patients had normal white blood cell counts and ESR [126]. Synovial fluid analysis varied with white cell counts ranging from 100 to 90,000/µL (mostly monocytes) and normal glucose. Alpert et al. reported that serum hypocomplementemia occurred in hepatitis-associated arthritis and was absent in uncomplicated acute hepatitis [130]. The low level of complements during the active phase of arthritis returns to normal as jaundice develops and joint symptoms resolve.

Low complement levels (found in 50% of cases) during acute illness, positive ANA (in approximately 10% of cases), positive rheumatoid factors (in approximately 25% of cases), or positive anti-CCP (in approximately 5%) can be present in HBV-associated arthritis, which clinically mimics other diagnoses, such as RA, systemic lupus erythematosus (SLE), drug reaction, etc., and may lead to misdiagnosis [111,127]. The detection of HBsAg, IgM anti-HBc antibodies, elevated AST/ALT levels, and the lack of joint destruction on radiographs can assist with the correct diagnosis. Arthritis is self-limited; symptoms resolve in 1–2 weeks without treatment. Non-steroidal anti-inflammatory drugs are typically effective in treating arthritis within a few days with no residual deformity recorded [2]. The resolution of arthritic symptoms happens during HBsAg clearance [129]. In two studies conducted by Lim et al. and Duffy et al., no chronic arthritis or recurrent arthritis was observed in 228 HBV-associated non-RA recruited patients and in 20 HBV-associated polyarthritis patients followed for 1–31 months, respectively [118,131].

### 7.3. Other Rheumatologic Diseases

Fibromyalgia is a common rheumatic complication during HBV infections. Ozsahin et al. and Yazmalar et al. reported that HBV-associated fibromyalgia developed in 22.0% of 77 HBV patients and 32.2% of 118 HBsAg-positive patients, respectively [124,132]. Fibromyalgia develops more often in patients with hepatitis B than in those without infection (32% vs. 5%) [127]. There was no difference in the incidence of this medical disorder in patients with untreated active chronic HBV, an inactive HBV infection, and patients receiving treatment for HBV [132].

In a systematic review and meta-analysis by Wang et. al, a lower rate of HBsAg positivity was found in patients with SLE than in the general population (OR = 0.28), indicating a lower frequency of HBV infection in SLE patients than patients in the control group [133]. A mechanism postulated as the pathogenesis of SLE is molecular mimicry in which a microbial pathogen may induce the development of autoantibodies [134]. In patients with HBV infection, it is possible that the virus also induces SLE in susceptible subjects [135]. Autoantibodies can develop to nuclear and cytoplasmic antigens, including dsDNA, histones, ribonucleoproteins, snRNP particle components, ribosomal proteins, and other cellular antigens [105]. An association of HBV infection with other rheumatologic diseases has been described in polymyalgia rheumatica, polymyositis, dermatomyositis, and Sjogren’s syndrome [136]. 

Treatment of rheumatic symptoms in patients with HBV infection is required in HBV patients with co-existent rheumatic diseases, such as SLE, RA, vasculitis, etc., [137]. It is important to determine whether these symptoms occur due to primary HBV chronic infection or due to a secondary process of rheumatic disease development since the treatment for HBV may decrease rheumatic symptoms and the treatment for rheumatic diseases with immunosuppression can cause increased viral replication [113]. According to the European and American Associations of Liver Diseases, the choices of antiviral drugs and the duration of treatment depend on the duration of immunosuppressive therapy and the status of the underlying HBV disease [138,139].

## 8. Dermatologic Manifestations

Several cutaneous manifestations have been reported in acute HBV infection, and these include urticaria, angioedema, and a varied spectra of rashes [140,141]. Kochar and Reddy studied 50 patients with HBV infection and found that the most frequent cutaneous complications were pruritus (42 patients, 92%) and urticaria/chronic urticaria (37 patients, 74%) [142]. Excluding spider nevi telangiectasia which occurs in 92%, another common vascular change was leukocytoclastic vasculitis which was found in 36%. Other extrahepatic cutaneous manifestations included erythema nodosum (28%), Gianotti–Crosti syndrome (12%), lichen planus (8%), pyoderma gangrenosum in two patients, and one patient with dermatomyositis-like illness. Several cutaneous manifestations are typically related to immune complex deposition [111,143]. 

### 8.1. Gianotti–Crosti Syndrome or Papular Acrodermatitis of Childhood

Hepatitis B virus is one of the most common causes of papular acrodermatitis of childhood. This skin disorder is characterized by rapid onset of small, flat, erythematous papules or papulovesicles, distributed on the face, distal extremities, and buttocks symmetrically [123,144]. The rashes can occasionally be very itchy and rarely involve the trunk, antecubital and popliteal surfaces, and mucous membranes. This complication usually resolves spontaneously in 1 to 6 weeks. It typically affects infants and young children, especially children between 2 and 6 years of age; it does not usually occur in adults [1]. During this cutaneous presentation, patients often have lymphadenopathy that can last for months in association with hepatomegaly, acute anicteric hepatitis, and HBsAg antigenemia [145,146]. In histology studies, mononuclear and histiocytic infiltrates around capillaries in the upper dermis are observed [147]. The new eruptions usually last a few days, but rashes can last up to 20 days. Anti HBV-Ab are not detected until 6 months after the resolution of papular acrodermatitis of childhood, and it has been reported that hepatitis is at its peak when the skin eruption fades away. 

### 8.2. Vasculitis

Palpable purpura, a cutaneous manifestation of small vessel vasculitis, is often associated with chronic HBV [143]. Another morphologic cutaneous vasculitis rash is the urticarial-like lesion, with leukocytoclastic vasculitis on histology and fibrinoid occlusion which results from immune complexes deposition and endothelial damage [1,148]. It occurs at 1 to 6 weeks before the icteric phase in approximately 15% to 20% of patients and spontaneously resolves. Initially, excess antigen generates soluble antigen–antibody complexes. The immune complexes contain HBsAg, IgG, IgM, and C3, and anaphylatoxins C3a and C5a, which cause urticarial vasculitis [141,149]. This type of rash can be associated with transient hypocomplementemia [140]. The clinical manifestations begin to subside when the antibody levels increase, and the immune complexes become less soluble and are easier to clear by the reticuloendothelial system.

### 8.3. Chronic Urticaria and Angioedema

Chronic spontaneous urticaria is a subtype of urticaria that lasts for more than 6 weeks. It occurs in 0.5–1% of the population. In a systematic review 5014 patients with chronic spontaneous urticaria, the prevalence of HBV infection in these patients was reported in 0–30% [150]. Up to 4% of patients present with angioedema [146]. Urticaria with periorbital edema has been reported as prodromal symptoms of acute hepatitis B infection [145]. 

### 8.4. Lichen Planus 

Lichen planus lesions can appear on the oral mucosa, tongue, and/or skin and are characterized by a lace-like white pattern. The definite prevalence of lichen planus in HBV patients is unknown. A study by Dogan with 88 hepatitis B carriers and 84 control patients reported a higher prevalence of oral lichen in patients with positive HBsAg than control patients (odd ratio = 8.3) [151]. Among lichen planus patients, 11–15% had chronic HBV hepatitis. In a study by Rebora, 44 patients with non-erosive lichen planus were investigated, and this study found that HBV-infected patients were two times more likely to develop lichen planus then the general population [152,153]. Approximately 4–9% patients with oral lichen planus tested positive for HBV infection [154,155]. Erosive oral lichen planus has been associated with chronic HBV infection [151]. However, a few studies have reported different results, and there is no clear evidence of the association of oral lichen planus [156].

### 8.5. Bullous Pemphigoid 

Bullous pemphigoid is an acquired autoimmune bullous skin disease that predominantly affects older individuals. A case report by Baykal et al. reported the development of bullous pemphigoid in a child one week after HBV immunization [157]. This case proposed that HBsAg can trigger these lesions by inducing a nonspecific immune reactivation or by stimulating a specific antibody production that may cross-react with bullous pemphigoid antigens.

### 8.6. Other Cutaneous Manifestations 

Other cutaneous disorders associated with HBV infections include erythema nodosum, erythema multiforme, pyoderma gangrenosum, lichenoid dermatitis, dermatomyositis-like syndrome, pitted keratolysis, and Raynaud’s phenomenon [127,141,151,158,159,160].

## 9. Conclusions

Hepatitis B virus infections can cause complex clinical syndromes, and the clinical presentation depends on whether the patient has an acute or chronic HBV infection. Acute HBV infections can cause fatigue, anorexia, nausea, vomiting, abdominal pain, and jaundice, and, in severe cases, acute liver failure. Chronic infections have a higher risk of hepatocellular carcinoma, cirrhosis, or fulminant liver failure, and this risk varies among the HBV genotypes. Direct acting antiviral drugs can prevent progressive liver disease in chronic hepatitis infections but usually do not cure it. 

Hepatic injury with inflammation and necrosis causes liver specific effects, such as decreased synthetic function and impaired clearance of metabolites, and systemic effects, including nonspecific symptoms, such as nausea, anorexia, vomiting, myalgias, arthralgias, and fever. These infections will lead to complicated immune responses, both humeral and cell-mediated immunity which can result in antibody formation with the development of immune complexes and cellular cytotoxicity secondary to CD8 cells. These responses can cause extrahepatic disorders, in particular cryoglobulinemia, glomerular disorders, and lymphocyte proliferation. A few studies have suggested that HBV can infect extrahepatic tissues and cause injury at these sites. In summary, clinicians should consider HBV infections in patients with a wide range of symptoms and clinical disorders. Testing for HBV in patients with extrahepatic disorders described in this review may identify unexpected infections and lead to antiviral treatment.

## Figures and Tables

**Table 1:** Table demonstrating each extrahepatic manifestation of HBV, pathophysiology, and treatment.

Extrahepatic Manifestations	Pathophysiology	Treatment
**Systemic diseases** Cryoglobulinemia	Circulating immune complexes and chronic antigen stimulation	1 Antiviral therapy with nucleoside analogs 2 Immunosuppressive agents, i.e., glucocorticoids 3 Plasma exchange 4 Rituximab
Polyarteritis nodosa	Immune dysregulation and immune complexes deposition	Antiviral therapy In severe cases, antiviral therapy and glucocorticoids ± plasma exchange
Serum sickness-like syndrome	Immune complexes lead to consumption of complement	Supportive treatment
**Hematologic** Lymphoma (NHL)	Direct-oncogenic effect mediated by intracellular HBV DNA Indirect-chronic stimulation of B-cell lymphocytes by extracellular HBV	Antiviral therapy and immunotherapy
Aplastic anemia	Bone marrow destruction by clonal expansion of cytotoxic T cells	Supportive treatment (blood transfusion) Definitive treatment-immunosuppressive therapy, allogenic bone marrow transplant
**Renal** Membranous nephropathy	Immune complex (HBsAg and Ab) deposition in the subepithelial space	Antiviral therapy, immunosuppressive
Membranoproliferative glomerulonephritis	Immune complex deposition in the mesangium	Not well established- a case report used antiviral therapy and glucocorticoids
IgA nephropathy	Viral transcription in the glomerular and renal tubular epithelial cells	Antiviral and immunosuppressive therapy
**Neurologic** Guillain-Barré Syndrome	Not well understood	Standard treatment with IVIG and antiviral therapy
Vasculitic neuropathy and non-vasculitic neuropathy	Immune complexes deposition in the vasa nervorum’s endothelium	Antiviral therapy, glucocorticoids, and rituximab
**Ophthalmic** Uveitis	Complement mediated immune process in the retinal vasculature	Antiviral therapy and supportive treatment
Optic neuritis	Not well understood	Not enough studies
**Rheumatologic** Rheumatoid arthritis	Immune complex deposition and production of autoantibodies	Antiviral therapy before immunosuppressive therapy

## Data Availability

Not applicable.

## Abbreviations

Anti-CCP: anti-cyclic citrullinated peptide; ALT, aminotransferase; DLBCL, diffuse large B-cell lymphoma; ESR, erythrocyte sedimentation rate; GBS, Guillain–Barré Syndrome; GN, glomerulonephritis; HAAA, hepatitis-associated aplastic anemia; HBV, hepatitis B virus; HBcAg, hepatitis B core antigen; HBeAg, hepatitis B e antigen; HBsAg, hepatitis B surface antigen; HCC, hepatocellular carcinoma; HCV, hepatitis C virus; IgA, immunoglobulin A; IgG, immunoglobulin G; IVIG, intravenous immunoglobulin; MN, membranous nephropathy; MPGN, membranoproliferative glomerulonephritis; NHL, non-Hodgkin lymphoma; PAN, polyarteritis nodosa; RA, rheumatoid arthritis; RF, rheumatoid factor; SLE, systemic lupus erythematosus.
